# Supplementary material for: Transcriptomic analysis to elucidate the response of honeybees (Hymenoptera: Apidae) to amitraz treatment
Source: PLoS One. 2020 Mar 6;15(3):e0228933. doi: 10.1371/journal.pone.0228933 (PMC7060074; doi:10.1371/journal.pone.0228933)
Supplement: Attachment — Submitted filename: Response to Reviewers.docx [file pone.0228933.s006.docx]

Response to PLOS ONE

Q: 1. We note the following previous request has not been addressed:

* We note that you are reporting an analysis of a microarray, next-generation sequencing, or deep sequencing data set. PLOS requires that authors comply with field-specific standards for preparation, recording, and deposition of data in repositories appropriate to their field. Please upload these data to a stable, public repository (such as ArrayExpress, Gene Expression Omnibus (GEO), DNA Data Bank of Japan (DDBJ), NCBI GenBank, NCBI Sequence Read Archive, or EMBL Nucleotide Sequence Database (ENA)). In your revised cover letter, please provide the relevant accession numbers that may be used to access these data.

A: Thank you for great suggestions. The sequencing data are available in the SRA database (SRR10595519-SRR10595524) of the NCBI system.

Response to Reviewers #1

1. In the part of Amitraz Treatment

Q: (1) the authors did not mention where was the Amitraz used in this experiment from, and lack of the neccessary information of the concentration or the status of purity of the chemical. Please add the related information.

A: Thank you for your suggests. We had added relevant information in the article.

Q: (2) In line 89. The authors conducted the experiment with the concentration of amitraz 9.4 mg/L, the problem is whether in this experiment, 9.4 mg/L of the amitraz is the sublethal doses to honeybees. Enven though one reference (Ref 24) listed in the manuscript has shown that in the previous study 9.4 mg/L of the amitraz is the sublethal doses to honeybees. For the reason that in this study, the amitraz maybe different with the chemical which the reference listed. Therefore, please add related information that amitraz 9.4 mg/L is truly sublethal to honeybees in this experiment.

A: It is a great suggests. Thank you! We had added the survival curve for treatment for the article (Fig. 1). Analysis this tab, we found that 9.4 mg/L of amitraz was truly sublethal to honeybees in this experiment.

Q: (3) The authors used expression of the concentration of amitraz both of “sublethal” and “subchronic”. Are there any difference between sublethal and subchronic?

A: Good suggest. Sublethal and subchronic are difference. Sublethal means having an effect less than lethal and is divided into acute and chronic. Subchronic means chronic sublethal. In article, the means of sublethal and subchronic was coinciding with reference.

Q: (4) In line 95-96, the authors treated the sample with the method “After 10 d, all bees were collected and placed at 4°C for 5 min to make them dizzy; the bees were then dissected on ice, and the midgut was removed and stored at −80°C”. Why did the authors not use liquid nitrogen to quickly ice the sample before the bees were dissected on ice and stored at -80°C?

A: Thank you for your suggests. We had used liquid nitrogen to quickly freeze the sample before the bees were dissected on ice and stored at -80°C, but not write in the article before and the detail information had been provided in materials and methods.

Q:  2. Line 155-157. “mapped reads were 27,182,392, 21,407,197, 24,815,509, 156 26,929,339, 28,752,629, and 157 27,107,676, respectively”. The number here are confusing, please correct them clearly.

A: Good suggestion. Thank you, This error has been corrected in article.

Q: 3. Authors choosed midgut and sequenced the midgut by transcriptomic methods to find the changes of the gene expression after exposed the honeybees in amitraz. If possible, please privide reasons why the authors choose the midgut as the target tissue. Some genes such as Antimicrobial peptides (AMPs) normally expressed higher in brain and hymolymph involved in the bees. Please disscussed them accordingly.

A: Midgut is an absorptive organ and the first affected organ for chemical compounds, additionally, the midgut epithelium is responsible for detoxification of ingested xenobiotics. We choose the midgut as the target tissue. AMPs genes normally expressed higher in brain and hymolymph involved in the honey bee, but in this study two AMP genes, encoding apidaecin (Apid1) and glycine-rich cell wall structural protein, were induced in amitraz-treated bees. The gene expression levels of AMPs will affect immunocompetence, therefore in our article we discussed the changes of AMPs gene expression in midgut.

Q: 4. Please provide the BUSCO analysis of the transcriptome to measure genome assembly and annotation completeness. Moreover, please provide the essential information of the Q20 percentage to show the quality of sequencing reads.

A: Thank you for great suggestions. All necessary informations about the Q20 percentage are provided in S2_Table.

Response to Reviewers #2

Q: 1. In the introduction part, the authors should provide more information about effects of amitraz on honey bees and more information regarding the function of midgut in honey bees. .

A: Great suggestions. Some detail information of the effects of amitraz and function of midgut on honeybees are provided in introduction.

Q: 2. RNA sequencing data must be deposited in NCBI Sequence Read Archive (SRA), and accession numbers must be provided in the paper.

A: Thank you for great suggestions. The sequencing data are available in the SRA database (SRR10595519-SRR10595524) of the NCBI system.

Q: 3. Lines 33-34, please specify upregulated or downregulated in honey bees exposed to amitraz?

A: Thank you for great suggestions. The specify DEGs are showed in Table S4 in results.

Q: 4. Line 64, the authors used midgut for transcriptome study, here is an example of a section of the intro that is relevant for the study. The authors should have expanded this part more.

A: We have expanded this part more.

Q: 5. Line 80, “a health colony”, how to define a colony is health or not? By the number of varroa mites in the colony or by virus titer of honey bees? Please specify more clearly.

A: Greet suggests. All honeybees were collected from a strong colony. All bee samples were strong and not infected with the parasite.

Q: 6. Line 85, bee bread or pollen? Bee bread means that the authors collected it directly from the comb.

A: We provided pollen to feed honeybees in this study and revised it inmethod part.

Q: 7. Line 94, why 10 days of treatment?

A: We are according to methods previously reported by Shi et al.(2017), and many studies research the effect of xenobiotics on honey bee by treated 10 days(O’Neal, Scott T et al., 2017; Du Yali et al., 2019)

Q: 8. Line 114, it seems that primer pairs in table 1 were used for validation of RNA-seq data, why authors list primer information here?

A: Sorry. Table 1 was appeared in the wrong place and we have revised it in article.

Q: 9. Line 126, why the authors didn’t use the latest version of representative genome of Apis mellifera (assembly Amel_HAv3.1)?
A: Sorry. Actually we truly use the latest version of representative genome of *Apis mellifera* (assembly Amel_HAv3.1) and we have revised it in article

Q: 10. Lines 139-140, why these genes were chosen for validating the RNA-seq data?
A: Firstly, the expression of these genes were significant different. Secondly, they were involved in the immunity and growth of honeybee.

Q: 11. Line, 141, it’s better to use two reference genes for qPCR analysis to obtain rigorous validation data.
A: Thank you for suggests. We had added an experiment by used other reference gene (*β-actin*) for qPCR analysis and showed in results.

Q: 12. Line 176, “23 most significantly differentially expressed genes”, these genes were selected by fold change or by p value? Also, the authors said adjusted p value or corrected p value were used for analysis, but in Supp Table, only p values were shown.
A: Thank you for suggests. Yes, these genes were selected by *p* value. We had added information and improved our Supp Table 5.

Q: 13. Line 199, did the authors do amplification efficiencies analysis before using the 2^−ΔΔCt^ method?

A: Yes, we did. Thank you.

Q: 14. Lines 226-236, this part of discussion is poor, and the authors just listed references, but didn’t discuss their data with previous studies well.
A: Thank you for great suggestions. The discussion had been enriched.

Q: 15. Line 252, “46 CYPs…”, again I suggest the authors should check the latest version of representative genome of Apis mellifera.
A: Thank you for great suggestions. We check the number, there are only 46 CYPs in genome of *A. mellifera* (Johnson 2012), and we do not found new CYP genes in honeybee at 2012 to 2019. However we revised the sentence to “Honey bee are known to have the far fewer number of CYP family genes than other insect.”

Q: 16. Overall, in the discussion part, the authors should discuss more about the biological significances of their data in honey bees. Also, the authors discuss little about GO function and KEGG Pathway analysis.
A: Great suggestions. The discussion part had been enriched

Q: 17. Figure 1 is the same to figure 2? Please check.

A: Sorry. The fig 1 was the same to fig 2, we deleted the fig 1 and replaced it with venn diagram.
