# Supplementary material for: Transcriptomic analysis to elucidate the response of honeybees (Hymenoptera: Apidae) to amitraz treatment
Source: PLoS One. 2020 Mar 6;15(3):e0228933. doi: 10.1371/journal.pone.0228933 (PMC7060074; doi:10.1371/journal.pone.0228933)
Supplement: Attachment — Submitted filename: Response to Reviewers.docx [file pone.0228933.s007.docx]

Response to Reviewers #1

Thank you for your comments.

Response to Reviewers #2

Q: 1. Page 5, lines 87-88, the first major methodological concern that I have concerns the use of just one colony for the study. I see this as a major flaw in the experimental design, as everybody working with social insects like honeybees should know that some replications is always needed at the colony level in addition to replication at the individual level. At least two or three colonies are always recommended for any type of study on social insects if the authors want to obtain results that can be generalized. In fact, all individuals within the same colonies are highly related genetically, and therefore there might be a specific effect of that colony affecting the results and the authors will not be able to identify these effects.

A: Great suggestions. Compared to treatment in only one colony, several colonies have a advantage of genetical diversity and avoid a specific effect of one colony. Some study researched the influence of environment stress above physiological response[1-3]. However, in the past decade several studies choose honey bees which was from same colony as samples [4-8]. Reference those studies, we explored the effect of amitraz on honeybees to by treat the honey bees from same colony.

Q: 2. Page 5, line 102, a second major methodological concern that I have concerns the use of acetone in amitraz-treated bees, but only sucrose–water solution was used in untreated bees. Amitraz was dissolved in acetone, so acetone-treated control bees should be used in the study. Therefore, acetone might have effects on gene expression of honey bees, and the authors will not be able to distinguish whether changing in gene expression is due to amitraz or acetone.

A: Thank you for great suggestions. We had acetone-treated control bees in the study, and revised in materials and methods.

Q: 3. Page 8, line 159, because 2−ΔΔCt method was used, please show amplification efficiency of each primer in the manuscript.

A: Thank you for great suggestions. We added amplification efficiency of each primer in Table 1

Q: 4. Page 13, line 240-241, only RPS5 was used?

A: Sorry, this a mistake here. We truly use two reference genes (*Rps5* and *β-actin*) for qPCR and we have revised it in article.

1.	Rutter L, Carrillo-Tripp J, Bonning BC, Cook D, Toth AL, Dolezal AG. Transcriptomic responses to diet quality and viral infection in Apis mellifera. BMC Genomics. 2019;20(1):412. Epub 2019/05/24. doi: 10.1186/s12864-019-5767-1. PubMed PMID: 31117959; PubMed Central PMCID: PMCPMC6532243.

2.	Zhu L, Qi S, Xue X, Niu X, Wu L. Nitenpyram disturbs gut microbiota and influences metabolic homeostasis and immunity in honey bee (Apis mellifera L.). Environ Pollut. 2019;258:113671. Epub 2019/12/20. doi: 10.1016/j.envpol.2019.113671. PubMed PMID: 31855676.

3.	Mondet F, Rau A, Klopp C, Rohmer M, Severac D, Le Conte Y, et al. Transcriptome profiling of the honeybee parasite Varroa destructor provides new biological insights into the mite adult life cycle. BMC Genomics. 2018;19(1):328. Epub 2018/05/08. doi: 10.1186/s12864-018-4668-z. PubMed PMID: 29728057; PubMed Central PMCID: PMCPMC5936029.

4.	Alburaki M, Karim S, Lamour K, Adamczyk J, Stewart SD. RNA-seq reveals disruption of gene regulation when honey bees are caged and deprived of hive conditions. The Journal of Experimental Biology. 2019;222(18). doi: 10.1242/jeb.207761.

5.	Christen V, Schirrmann M, Frey JE, Fent K. Global Transcriptomic Effects of Environmentally Relevant Concentrations of the Neonicotinoids Clothianidin, Imidacloprid, and Thiamethoxam in the Brain of Honey Bees ( Apis mellifera). Environ Sci Technol. 2018;52(13):7534-44. Epub 2018/06/01. doi: 10.1021/acs.est.8b01801. PubMed PMID: 29851480.

6.	Shi TF, Wang YF, Liu F, Qi L, Yu LS. Sublethal Effects of the Neonicotinoid Insecticide Thiamethoxam on the Transcriptome of the Honey Bees (Hymenoptera: Apidae). J Econ Entomol. 2017;110(6):2283-9. Epub 2017/10/19. doi: 10.1093/jee/tox262. PubMed PMID: 29040619.

7.	Emery O, Schmidt K, Engel P. Immune system stimulation by the gut symbiont Frischella perrara in the honey bee (Apis mellifera). Mol Ecol. 2017;26(9):2576-90. Epub 2017/02/17. doi: 10.1111/mec.14058. PubMed PMID: 28207182.

8.	Mao W, Schuler MA, Berenbaum MR. Disruption of quercetin metabolism by fungicide affects energy production in honey bees (Apis mellifera). Proc Natl Acad Sci U S A. 2017;114(10):2538-43. Epub 2017/02/15. doi: 10.1073/pnas.1614864114. PubMed PMID: 28193870; PubMed Central PMCID: PMCPMC5347564.
