# Supplementary material for: Transcriptomic analysis to elucidate the response of honeybees (Hymenoptera: Apidae) to amitraz treatment
Source: PLoS One. 2020 Mar 6;15(3):e0228933. doi: 10.1371/journal.pone.0228933 (PMC7060074; doi:10.1371/journal.pone.0228933)
Supplement: Attachment — Submitted filename: Response to Reviewers.docx [file pone.0228933.s008.docx]

Response to Reviewers #2

Q: 1. Lines 87-88, ‘a healthy colony’ should be ‘an apparently healthy colony’.

A: Thank you for great suggestions. We have revised it in article

Q: 2. Lines 153, where is the total RNA for qPCR validation from? How many replicates were used for qPCR validation? More details needed here.

A: Great suggestions. The total RNA for qPCR is the same as the total RNA for RNA-seq. We used three replicates for qPCR validation. We have added relevant information in the article.

Q: 3. Line 157, for Table 1, the author should cite references if primer sequences were from published papers. Also, please check the primer sequences carefully, the reverse primer sequences of actin is wrong.

A: Sorry. The reverse primer sequences of actin is wrong, we have revised it and cite references in article.

Q: 4. Line 181, ‘…were calculated using result in S2 Table’? Please check and rephrase the sentence.

A: Thank you for good suggestions. We have rephrased this sentence in article.

Q: 5. Line 224, I got confused with the qPCR analysis result, the authors calculated the expression level of the gene of interest using RPS5 and actin respectively, and the qPCR data was shown in fig5a and 5b respectively. Actually, the authors should calculate the geometric mean of references genes first and then calculate the expression level of each target gene based on the geometric mean of references genes. Please refer to (Garrido et al., 2013; Wu et al., 2017).

A: Thank you for great suggestions. Refer to this way, we make a new fig to show our qPCR results.

Q: 6. Lines 260-262, Please cite references here. Also, should be “… in different developmental stages of honey bees”.

A: Great suggestions. We have revised it and add relevant information in the article.

Q: 7. Lines 262-264, this sentence just repeats the content of lines 260-262 and is redundant. Please delete or rephrase the sentence.

A: It is a great suggests. Thank you! We have rephrased the sentence in the article.

Q: 8. Line 265, ‘Thiamethoxam treated honey…exposed to either thiamethoxam…’, again further treated with the same pesticide? Please check.

A: Thank you for great suggestions. We check this referenc, they again further treated with the same pesticide truly.

Q: 9. Lines 265-266, gene names and Nosema should be printed in italics.

A: Good suggestions. We have revised it in article

Q: 10. Line 267, why the authors conclude that glycine-rich cell wall structural protein is antimicrobial peptides.

A: Sorry, we have revised it in article

Q: 11. Lines 287-290, the content regarding phytochemical metabolism, furanocoumarins, and quercetin is not closely related to the topic of this study. Please delete.

A: Thank you for great suggestions. We have deleted it in article

Q: 12. Line 297, ‘…were significantly upregulated in susceptible strain…’? Please specify.

A: It is a great suggests. Thank you! We have specified it in article.

Q: 13. As I mentioned earlier, the authors should discuss more about GO function and KEGG pathway analysis in the discussion part.

A: Great suggestions. The discussion part had been enriched

Q: 14. The author should incorporate the response to reviewer comments into the revised manuscript. For example, the justification for only one colony was used for sample preparation in the study.

A: Great suggestions. We have incorporated the response to reviewer comments into the revised manuscript.
